# Multiscale Porous Carbon Materials by In Situ Growth of Metal–Organic Framework in the Micro-Channel of Delignified Wood for High-Performance Water Purification

**DOI:** 10.3390/nano13192695

**Published:** 2023-10-03

**Authors:** Youngho Jeon, Dabum Kim, Suji Lee, Kangyun Lee, Youngsang Ko, Goomin Kwon, Jisoo Park, Ung-Jin Kim, Sung Yeon Hwang, Jeonghun Kim, Jungmok You

**Affiliations:** 1 Department of Plant & Environmental New Resources, Kyung Hee University, 1732 Deogyeong-daero, Giheung-gu, Yongin-si 17104, Republic of Koreacrew75@khu.ac.kr (S.Y.H.); 2 Department of Chemical and Biomolecular Engineering, Yonsei University, 50 Yonsei-ro, Seodaemun-gu, Seoul 03722, Republic of Korea

**Keywords:** multiscale porous carbon, tempo oxidation, water purification, wood microchannel

## Abstract

Porous carbon materials are suitable as highly efficient adsorbents for the treatment of organic pollutants in wastewater. In this study, we developed multiscale porous and heteroatom (O, N)-doped activated carbon aerogels (CAs) based on mesoporous zeolitic imidazolate framework-8 (ZIF-8) nanocrystals and wood using 2,2,6,6-tetramethylpiperidine-1-oxyl (TEMPO) oxidation, in situ synthesis, and carbonization/activation. The surface carboxyl groups in a TEMPO-oxidized wood (TW) can provide considerably large nucleation sites for ZIF-8. Consequently, ZIF-8, with excellent porosity, was successfully loaded into the TW via in situ growth to enhance the specific surface area and enable heteroatom doping. Thereafter, the ZIF-8-loaded TW was subjected to a direct carbonization/activation process, and the obtained activated CA, denoted as ZIF-8/TW-CA, exhibited a highly interconnected porous structure containing multiscale (micro, meso, and macro) pores. Additionally, the resultant ZIF-8/TW-CA exhibited a low density, high specific surface area, and excellent organic dye adsorption capacity of 56.0 mg cm^−3^, 785.8 m^2^ g^−1^, and 169.4 mg g^−1^, respectively. Given its sustainable, scalable, and low-cost wood platform, the proposed high-performance CA is expected to enable the substantial expansion of strategies for environmental protection, energy storage, and catalysis.

## 1. Introduction

With the rapid development of human society, the world has become faced with severe environmental pollution issues caused by hazardous organic chemicals, such as dyestuffs, oils, and organic solvents [1,2,3]. Particularly, industrial waste affects biodiversity, ecosystems, and human health through soil and water contamination. Organic dye chemicals are widely used in essential industries, such as dye, plastic, textile, paper, pharmaceutical, and food [4,5,6,7,8,9], and although most toxic organic dyes are filtered from industrial waste using water purification technology, their leakage into water bodies, even at low concentrations, can lead to serious environmental problems and hazards to human health. Particularly, their non-biodegradability in water, thermal stability, and use in everyday life increase their harmful effects [10,11,12]. Thus, wastewater treatment is essential to protect ecosystems and humans from harmful water pollutants. To prevent an increase in the environmental hazards caused by organic waste, various wastewater treatment approaches, such as coagulation, filtration, precipitation, adsorption, ion exchange, and advanced oxidation processes, have been employed for the removal of organic dyes from water systems [13,14,15,16,17,18,19,20]. Among them, the most common technique is adsorption based on the use of adsorbents (e.g., zeolite, clay, and silica) with high specific surface areas [21,22,23,24,25,26]. Adsorption is a relatively simple, effective, and economical technique (based on materials and operation cost and the minimal production of secondary pollutants). Thus, it is highly desirable to develop a high-performance absorbent for treating organic pollutants.

Carbon aerogels (CAs), which exhibit a large surface area, low density, and high porosity, have recently attracted considerable attention in environmental treatment, energy storage, catalysis, and sensors fields [27,28,29,30]. Over the past decades, CAs have been fabricated using (1) nanocarbon units (carbon nanotubes, carbon nanofibers, and graphene nanosheets) and (2) polymerized organic gels derived from precursors [31,32,33,34,35]. However, despite their intrinsic benefits, their high manufacturing cost and complex manufacturing processes, which hinder their large-scale commercial production, have limited their further application. Furthermore, most precursors used for the fabrication of CAs are considered to be carcinogenic to humans. To address these limitations, several recent studies have reported the high adsorption capability of biomass-derived CAs for organic pollutants [36,37,38,39]. For example, Yamauchi et al. reported the large-scale synthesis of biomass polymer-derived CAs using a simple and sustainable method as a high-performance adsorbent [40]. They demonstrated that biomass material is a suitable building block for CAs owing to its natural abundance, sustainability, and cost-effectiveness. Additionally, they demonstrated the enhanced surface area and mechanical properties of nanoparticle-embedded CAs, thereby expanding the applications of CAs. 

Wood, one of the most abundant natural biomasses, exhibits a hierarchical structure with parallel hollow tubes comprising cellulose nanofibers and an amorphous matrix of lignin and hemicellulose [41,42,43]. As a biomass material, wood exhibits multiscale porous structures ranging from the macro to nano levels [44]. Macroscopic pores, such as cell lumen, resin channels, and pit apertures, as well as mesoporous pores, such as pores in the cell wall, can provide large specific surface areas and strong adsorption capacities. This hierarchically porous structure along with its natural abundance makes it an ideal template for the development of functional green nanocomposites [45]. Metal–organic frameworks (MOFs) are crystalline compounds, containing three-dimensional (3D) networks of metal ions and organic linkers, with tunable porosity [46,47]. Recently, MOFs have been extensively investigated for separation, storage, catalysis, and sensing applications, owing to their high specific surface area, large porosity (micropores/mesopores), and low density [48,49,50,51,52,53,54,55,56,57]. However, despite the innovative utilization of MOFs, most MOFs are produced in the form of crystals and powders, which limit their use in real-world applications. Recently, several studies have demonstrated the successful synthesis of wood/MOF composite adsorbents by the in situ synthesis of MOFs within the aligned wood channels [36,37,58,59]. For example, He et al. developed ZIF-67@wood aerogel via the in situ wet chemical deposition of ZIF-67 on the wood aerogel for the adsorptive purification of polluted wastewater [36]. However, the lack of continuous nucleation sites in natural wood templates results in a low mass loading and poor uniformity of MOFs, which significantly affect the adsorption efficiency of these adsorbents. To the best of our knowledge, there is only one report (Zhou et al. described a foam-like TEMPO-oxidized wood/MOF aerogels for efficient CO_2_ capture) describing the use of a TEMPO-oxidized wood template for the in situ growth of MOFs [60]. 

Herein, we describe the development of high-performance activated CAs with a hierarchical pore structure ranging from the micro to meso- and macroscale by harnessing the advantages of wood and MOFs. To the best of our knowledge, this is the first report on a wood/ZIF-8-derived CA for the efficient removal of organic pollutants. In this study, a 2,2,6,6-tetramethylpiperidine-1-oxyl (TEMPO)-oxidized wood (TW) template with surface carboxyl groups was utilized as an important building block for the CAs. The surface carboxyl groups in the TW enabled the coordination of the cellulose network with multivalent metal ions, resulting in the efficient nucleation and in situ growth of MOFs within the wood template. Additionally, ZIF-8 with a high specific surface area and nitrogen content was synthesized in situ inside the TW, after which the wood was subjected to a freeze-drying process to obtain ZIF-8/TW-A. Subsequently, the ZIF-8/TW-A was subjected to a direct carbonization/activation process to obtain a highly interconnected multiscale porous and O, N-doped CA (denoted by ZIF-8/TW-CA), where wood with a hierarchical cellular structure served as a template, and ZIF-8 provided micro and mesoporous networks. The resultant ZIF-8/TW-CA exhibited a low density, high specific surface area, and outstanding adsorption capacities for organic pollutants.

## 2. Materials and Methods

### 2.1. Materials

Balsa wood (Ochroma pyramidale) was purchased from Balsanara Co. (Chungju, Chungcheong-do, Republic of Korea). In addition, 6% sodium hypochlorite (NaClO) solution, methanol, *tert*-butanol, ethanol, *n*-hexane, chloroform, sodium hydroxide (NaOH), and hydrochloric acid (HCl) were purchased from Duksan Pure Chemicals Co. Ltd., Ansan, Gyeonggi-do, Republic of Korea. Zinc nitrate (Zn(NO_3_)_2_·6H_2_O_6_), TEMPO, Sudan III, rhodamine B (RhB), phosphate-buffered saline (PBS), and sodium chlorite (NaClO_2_) were purchased from Sigma-Aldrich, and 2-methyl imidazole (2-Melm) was purchased from TCI Co., Chou-ku, Tokyo, Japan).

### 2.2. Preparation of the TEMPO-Oxidized Wood

First, the balsa wood was cut into the desired size and immersed in 6% NaClO solution with vacuum impregnation for 24 h. After delignification, the delignified wood (DW) was washed with deionized water (DIW), and the lignin contents of the DW were measured using the Klason method. Briefly, the dry mass (0.3 g, M_0_) was measured and treated with H_2_SO_4_ (72%, 3 mL) for 1 h at 30 °C. Thereafter, the mixtures were transferred to a beaker, diluted to 4% by mass of H_2_SO_4_ by adding 84 mL of DIW, and then boiled in an autoclave (121 °C, 1 h). After cooling, the mixtures were filtered using a glass filter membrane and washed with 250 mL of DIW water. The insoluble materials were dried and weighed (M_d_), and the lignin content was calculated as follows: (M_d_/M_0_) × 100%. For TEMPO oxidation, the DW was immersed in TEMPO/NaClO/ NaClO_2_ (0.016 g/1.25 mL/1.13 g) dissolved in PBS solution (100 mL, 0.1 M, pH 7.4) at 60 °C for 48 h. After the TEMPO oxidation, the TWs were washed in an ethanol/DIW mixture (1:1, *v*/*v*) and stored in methanol.

### 2.3. Preparation of ZIF-8/TW-A

ZIF-8/TW-A was synthesized according to a previously reported procedure [61]. First, the TW was immersed for 8 h with vacuum infiltration to achieve ion exchange between Zn and Na ions in Zn(NO_3_)_2_·6H_2_O_6_ solution, which was prepared by dissolving 2.4 g of Zn(NO_3_)_2_·6H_2_O in 20 g of methanol and 3 g of DIW. Subsequently, the 2-Melm solution (13.2 g of 2-Melm in 20 g of methanol and 3 g of DIW) was poured into the aforementioned solution. After 24 h, the ZIF-8/TW was washed with methanol, and the solvent was exchanged with tert-butanol. Thereafter, the ZIF-8/TW was subjected to freeze-drying.

### 2.4. Preparation of ZIF-8/TW-CA

First, the ZIF-8/TW-A composites were carbonized at 250 °C for 1 h in a tube furnace to remove water from the sample. Thereafter, the temperature was increased to 850 °C for 3 h in an N_2_ atmosphere at a heating rate of 5 °C/min. After carbonization, the samples were further heated to 350 °C for 1 h in air at a heating rate of 5 °C/min. For comparison, ZIF-8/DW-CA samples were prepared without TEMPO oxidation, using the same process as that used for ZIF-8/TW-CA.

### 2.5. Characterizations

The morphology of the ZIF-8/wood-based CA was investigated using field emission scanning electron microscopy (FE-SEM; Carl Zeiss, model AURIGA, Oberkochen, Germany), and elemental analysis was performed using energy-dispersive X-ray spectroscopy (EDS). The thickness of the samples was measured using a digital caliper (Mitutoyo Co., model 2109S-10, Kawasaki, Japan). The argon adsorption isotherms were analyzed using a Micromeritics ASAP 2010 micropore system and calculated using the Brunauer–Emmett–Teller (BET) and density functional theory (DFT) method. Fourier transform infrared (FT-IR) spectroscopy data were recorded from 4000 to 400 cm^−1^ using a Spectrum One System FT-IR spectrometer (Perkin-Elmer, Waltham, MA, USA). The chemical composition of the surface elements from 0 to 1200 eV was investigated using X-ray photoelectron spectroscopy (XPS; Thermo Electron model, K-Alpha, Waltham, MA, USA), and the crystallinity of the samples was analyzed using X-ray diffraction (XRD; Bruker, model D8 Advance, Billerica, USA) in the scanning range of 3−60°. Raman spectra were recorded using a spectrometer (QE-PRO, Ocean Optics, Inc., OR, USA) equipped with a 785 nm (22 mW) laser. Thermogravimetric analysis (TGA) was performed using a Discovery TGA (TA Instruments, New Castle, USA) under nitrogen atmosphere at a heating rate of 10 °C min^−1^. Ultraviolet–visible (UV–VIS) curves were obtained using a UV–VIS spectrophotometer (Shimadzu, model UV-3600 Plus, Nishinokyo, Japan).

### 2.6. Adsorption Studies

The adsorption properties of ZIF-8/TW-CA were investigated via batch adsorption experiments with RhB. All experiments were performed by immersing 10 mg of the adsorbent samples in 10 mL of each concentration (10–1000 mg L^−1^, pH 7) of RhB under stirring using a shaker at 80 rpm for 24 h. Thereafter, 1 M HCl and 1 M NaOH were added to the dye solutions to adjust the pH. The concentration of the solution after adsorption was analyzed using a UV–VIS spectrophotometer by measuring the prominent wavelength at 553 cm^−1^. The adsorption spectra and standard calibration curves of the different concentrations of the RhB solution are illustrated in Figure S4. To evaluate the recyclability of the CA, the adsorbed adsorbent was rinsed thrice in a solution mixture containing ethanol and DIW (1:1, *v*/*v*) to desorb RhB. After washing, the sample was immersed in 10 mg L^−1^ of RhB again. The adsorption capacity (*q_e_*) for RhB and the removal efficiency (%) were calculated as follows:

(1) $$q_{e}=\left(C_{0}-C_{e}\right)V/m,$$

(2) $$Removal\;efficiency\;\left(\%\right)=\left(C_{0}-C_{e}\right)/C_{0}\times 100,$$

where *q_e_* (mg g^−1^) is the quantity of RhB adsorbed at equilibrium; *C_0_* (mg L^−1^) is the initial concentration of RhB; *C_e_* (mg L^−1^) is the residual concentration of RhB solution after adsorption; and *V* (L) and *m* (g) are the volume of the adsorbate and mass of the adsorbent, respectively. 

#### Adsorption Models

To understand the nonlinear forms of the kinetic adsorption and isotherm models, four adsorption models were adopted (isotherm model, nonlinear Langmuir (3) and nonlinear Freundlich (4); kinetic model, nonlinear pseudo-first-order (5) and nonlinear pseudo-second-order (6)). The equations and parameters of the adsorption models were expressed as follows: [62]

(3) $$q_{e}=q_{max}K_{L}C_{e}\;/\left(1+K_{L}C_{e}\right),$$

(4) $$q_{e}=K_{F}C_{e}^{1/n},$$

(5) $$q_{t}=q_{e}\left(1-e^{-k_{1}t}\right),$$

(6) $$q_{t}=q_{e}^{2}K_{2}t/\left(1+q_{e}K_{2}t\right).$$

## 3. Results

**Figure 1:**
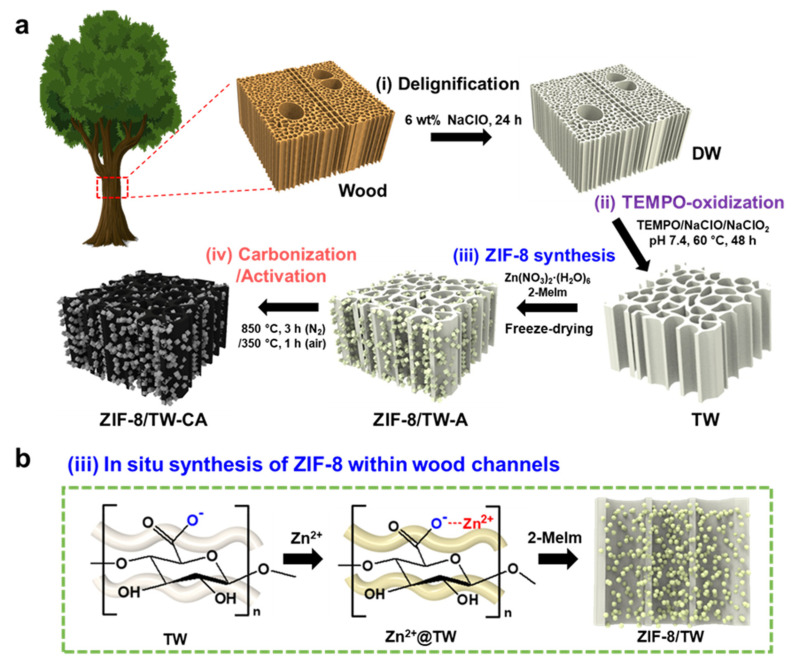
(**a**) Schematic representation of the fabrication of ZIF-8/TEMPO-oxidized wood carbon aerogel (ZIF-8/TW-CA): (i) delignification using NaClO solution (6 wt%, 24 h); (ii) TEMPO oxidation using TEMPO/NaClO/NaClO_2_ solution (pH 7.4, 60 °C, 48 h) (purple); (iii) in situ synthesis of ZIF-8 using Zn(NO_3_)_2_, 2-MeIm solution, and freeze-drying (blue); (iv) carbonization at 850 °C (3 h, N_2_ atmosphere) and activation at 350 °C (1 h, air) (red). (**b**) Illustration of the in situ process for obtaining ZIF-8/wood composites via TEMPO-oxidization.

Figure 1a shows a schematic representation of the fabrication of ZIF-8/TW-CA through delignification, TEMPO oxidation, in situ ZIF-8 synthesis, and carbonization processes. First, pristine woods were pretreated via delignification to enhance the porosity and chemical accessibility of the woods (Figure 1a(i)) [63]. After the pretreatment, the yellowish color of the wood changed into white, indicating the delignification of the wood (Figure S1a,b). The successful delignification of the wood was evaluated using the Klason method and FT-IR (Figure S2). The results reveal a reduction of approximately 90% in the lignin content of the DW (2.42%) compared to that of pristine wood (21.5%) (Figure S2a). Furthermore, the decrease in the lignin-specific IR peaks at 1590, 1501, and 1452 cm^−1^, which can be attributed to aromatic skeletal vibration, and that at 1230 cm^−1^, corresponding to the guaiacyl ring with C–O stretching, confirmed the removal of lignin from the wood (Figure S2b). Additionally, the decrease in the intensity of the carbonyl group (C=O) at 1737 cm^−1^ indicated the removal of hemicellulose from the wood channel and middle lamella [64,65]. After delignification, we conducted TEMPO-mediated oxidation to selectively convert C6 primary hydroxyl groups to carboxyl groups on the surface of cellulose microfibrils (Figure 1a(ii) and Figure S1c) to provide nucleation sites for the subsequent in situ ZIF-8 synthesis (Figure 1b). Conductometric titration revealed that the carboxyl group content in the TW was 1.68 mmolg^−1^. After complete washing, the TWs were immersed in a zinc nitrate solution. The cell walls of the wood efficiently adsorbed Zn^2+^ via the electrostatic interactions between Zn^2+^ and the carboxylic groups. After methanol washing, the Zn^2+^-loaded TWs were transferred into an organic ligand solution to achieve the nucleation and growth of ZIF-8 nanoparticles (Figure 1a(iii),b). Thereafter, the sample was freeze-dried to obtain ZIF-8/TW-A (Figure S1d), after which ZIF-8/TW-A was carbonized into ZIF-8/TW-CA under a N_2_ atmosphere at 850 °C and further activated at 350 °C in air (Figure 1a(iv) and Figure S1e). After carbonization, the air activation as a physical activation can effectively improve the surface area of the hierarchical porous structure and increase the number of oxygen-containing functional groups on the carbon surface in the ZIF-8/TW-CA [66].

**Figure 2:**
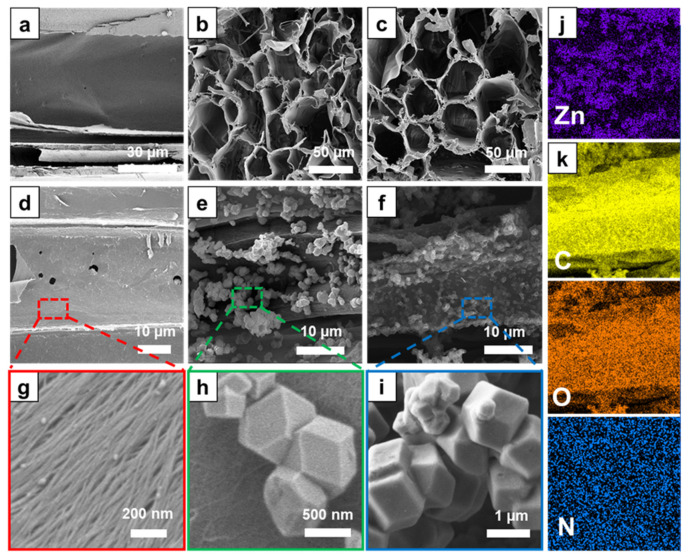
Field emission scanning electron microscopy (FE-SEM) images of (**a**,**b**) balsa wood, (**c**,**d**,**g**) tempo-oxidized wood (TW) (red), (**e**,**h**) ZIF-8/TW-A (green), and (**f**,**i**) ZIF-8/TW-CA (blue). Corresponding energy-dispersive spectroscopy (EDS) mapping images of (**j**) ZIF-8/TW-A and (**k**) ZIF-8/TW-CA.

The FE-SEM images (Figure 2a–c) indicate that the pristine wood and TW exhibited a typical natural wood structure with honeycomb-like cellular structures and vertical channels, confirming that pretreatments, such as delignification and TEMPO oxidation, did not result in the structural collapse of the wood. Additionally, mesopores (2–50 nm) and macropores (>50 nm) were observed at the aligned cellulose fibers in the cell wall of TW (Figure 2d,g), which may be attributed to the delignification and TEMPO oxidation. The extensive loss of lignin and its conversion into carboxyl groups during delignification and TEMPO oxidation enhanced the cell wall porosity and increased the specific surface area. The enhanced porosity and surface area will induce good chemical accessibility for the subsequent in situ ZIF-8 synthesis. Figure 2e,h demonstrate the good deposition of ZIF-8 on the entire surface of the cell walls inside the TW without large aggregation. After carbonization, ZIF-8/TW-A was converted into ZIF-8/TW-CA. Although the size of the ZIF-8-derived nanoporous carbon particles decreased because of the decomposition of organic ligands, the final ZIF-8/TW-CA adequately retained the original ZIF-8 shape (Figure 2f,i). The carbonization temperature is one of the critical parameters that determines the surface area and pore volume of the nanoporous carbon [67]. Additionally, after carbonization, the ZIF-8-embedded wood composites were converted into interconnected, heteroatom-doped, and porous CA by harnessing the advantages of ZIF-8 and wood [40]. 

**Figure 3:**
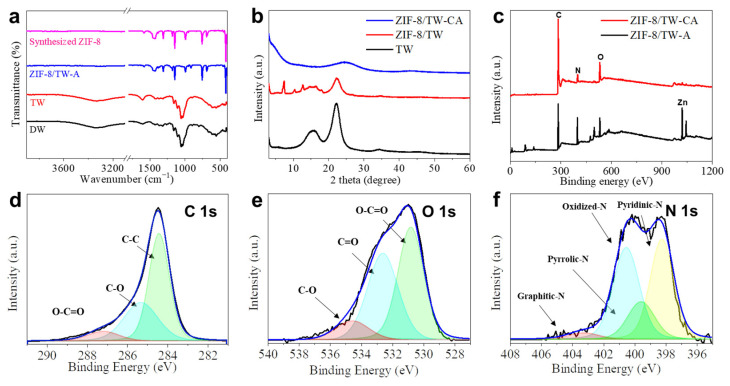
(**a**) Fourier transform infrared (FT-IR) spectra of delignified wood (DW), TW, ZIF-8/TW-A, and ZIF-8 nanoparticles. (**b**) X-ray diffraction (XRD) pattern of TW, ZIF-8/TW-A, and ZIF-8/TW-CA. (**c**) X-ray photoelectron spectroscopy (XPS) profiles of ZIF-8/TW-A and ZIF-8/TW-CA and high-resolution (**d**) C 1s, (**e**) O 1s, and (**f**) N 1s XPS profiles of ZIF-8/TW-CA.

Heteroatom-doped carbon is well known to exhibit excellent adsorption capabilities [68]. Compared to that of ZIF-8/TW-A, the volume of ZIF-8/TW-CA reduced by approximately 40%. Additionally, ZIF-8/TW-CA exhibited a low density of 56 mg cm^−3^. The FT-IR results demonstrate the successful chemical transformation during the fabrication of ZIF-8/TW-CA (Figure 3a). A strong IR peak of C=O carbonyl groups was observed in the FT-IR spectrum of TW at 1603 cm^−1^, confirming the TEMPO oxidation of pristine wood. Additionally, the representative IR peaks of neat ZIF-8 nanoparticles at 1584 cm^−1^, which is assigned to the C=N stretching, and within 1350–1500 cm^−1^, which is attributed to the ring stretching in imidazole, were observed in the FT-IR spectrum of ZIF-8/TW-A. 

This suggests the successful in situ growth of ZIF-8 in the TW. The XRD pattern further confirmed the embedding of ZIF-8 into the TW template (Figure 3b and Figure S3), in which two broad diffraction peaks were observed at approximately 14.8°–16.8° and 22.5°, indicating the presence of cellulose I crystals in the wood. In addition, two broad diffraction peaks assigned to cellulose were observed in the XRD pattern of ZIF-8/TW-A, along with diffraction peaks at 7.2, 12.7, 16.3, and 18.4°, which can be attributed to the (110), (211), (310), and (222) crystal faces of ZIF-8, respectively (Figure S3a), demonstrating the successful growth of ZIF-8 crystals. These XRD patterns are consistent with the FT-IR results (Figure 3a). Further, two broad peaks were observed in the XRD pattern of ZIF-8/TW-CA at 25 and 43°, which are characteristic peaks of the (101) and (002) planes of carbon, respectively (Figure S3b) [40,60,66]. Next, we examined the chemical composition and environment of ZIF-8/TW-A and ZIF-8/TW-CA using XPS (Figure 3c). Compared to ZIF-8/TW-A (prior to carbonization), ZIF-8/TW-CA did not exhibit a Zn-related signal peak, suggesting the efficient removal of Zn content via carbonization. In addition, the XPS profiles revealed that ZIF-8/TW-CA comprised C, O, and N elements. Three peaks were observed in the high-resolution C 1s spectrum (Figure 3d) at 284.4, 285.4, and 287.2 eV, which can be attributed to the characteristic signals of C–C, C–O, and O–C=O bonds, respectively [66]. Additionally, three peaks were observed in the XPS O 1s spectrum (Figure 3e) at 530.8, 532.6, and 534.5 eV, which can be attributed to O–C=O, C=O, and C–O bonds, respectively [69]. Four peaks were observed in the N 1s spectrum (Figure 3f) at 398.3, 399.5, 400.5, and 403.2 eV, which can be attributed to pyridinic-N, pyrrolic-N, oxidized-N, and graphitic-N, respectively [40]. The introduction of heteroatoms (e.g., N, O) into the CA increased its adsorption ability for organic pollutants [70]. The XPS results of ZIF-8/TW-A and ZIF-8/TW-CA are with the EDS results (Figure 2j,k).

**Figure 4:**
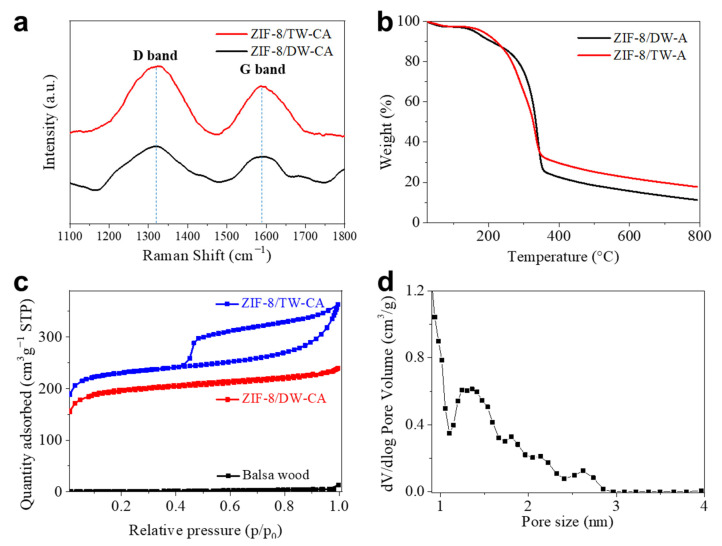
(**a**) Raman spectra of ZIF-8/TW-CA and ZIF-8/DW-CA. (**b**) Thermal stability of the ZIF-8/wood composite aerogels. Thermogravimetric analysis (TGA) curve of ZIF-8/DW-A and ZIF-8/TW-A under nitrogen atmosphere. (**c**) Argon adsorption/desorption isotherms of ZIF-8/wood carbon aerogel composites. (**d**) The corresponding pore-size distributions of ZIF-8/TW-CA.

The Raman spectra (Figure 4a) of ZIF-8/DW-CA and ZIF/TW-CA exhibited two characteristic carbon peaks at 1327 (D: disordered carbon) and 1588 (G: graphitic carbon). The ID/IG ratios of ZIF-8/DW-CA and ZIF/TW-CA were 1.37 and 1.24, respectively, indicating that ZIF-8/TW-CA exhibited larger defects than ZIF-8/DW-CA, which can be attributed to the high degree of N-doping [40,66]. Next, thermogravimetric analysis (TGA) was conducted to investigate the thermal properties and compare the ZIF-8 loading amounts of the ZIF-8/wood aerogels (Figure 4b). The decomposition of the ZIF-8/wood aerogels was observed to begin at approximately 240 °C, which can be attributed to the thermal degradation of the cellulose content. The residual content of ZIF-8/TW-A (20%) was higher than that of ZIF-8/DW-A (12%), indicating the higher ZIF-8 loadings of ZIF-8/TW-A. This difference between their ZIF-8 loadings can be attributed to the different amounts of carboxyl groups from the TEMPO oxidation. To examine the specific surface area and pore-size distribution, we conducted argon adsorption–desorption measurements for the pristine wood, ZIF-8/DW-CA, and ZIF-8/TW-CA. The pristine wood hardly adsorbed argon and only slightly adsorbed argon towards the end of the measurement, indicating that the wood was only composed of macropores (Figure 4c and Table 1). 

Compared to those of ZIF-8/DW-CA (662.1 m^2^ g^−1^, 0.30 cm^3^ g^−1^), ZIF-8/TW-CA exhibited a large specific surface area (785.8 m^2^/g) and pore volume (0.46 cm^3^ g^−1^). These results confirm that the high ZIF-8 loading mass owing to the TEMPO oxidation promoted the nanoporous structure of the wood-based CA. The pore-size distribution range of ZIF-8/DW-CA and ZIF-8/TW-CA was 0.8–2.8 nm, indicating that carbonization and activation processes led to the presence of micro- and mesopores in both wood-based CAs because of ZIF-8 and chemical activation process. (Figure 4d and Figure S5). Next, the adsorption ability of ZIF-8/TW-CA was investigated using RhB, which is a model dye.

**Figure 5:**
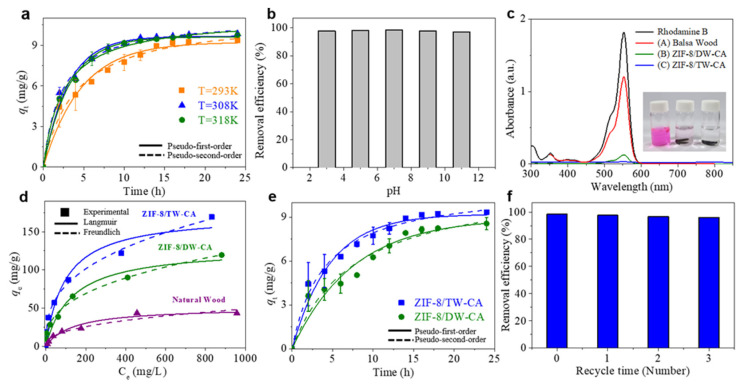
(**a**) Adsorption kinetic of RhB on ZIF-8/TW-CA at 293, 308, and 318 K fitted using pseudo-first-order and pseudo-second-order kinetic models. (**b**) Removal efficiency of RhB (10 mg/L) for ZIF-8/TW-CA in the pH range of 3–11. (**c**) Absorbance spectra of pristine wood, ZIF-8/DW-CA, and ZIF-8/TW-CA for RhB solution (10 mg L^−1^). (**d**) Adsorption isotherms of pristine wood, ZIF-8/DW-CA, and ZIF-8/TW-CA at various RhB concentrations in water (Ce) fitted using Langmuir and Freundlich models. (**e**) Adsorption kinetic of RhB (10 mg L^−1^) exposed to ZIF-8/DW-CA and ZIF-8/TW-CA fitted using pseudo-first-order and pseudo-second-order kinetic models. (**f**) Recycling ability of ZIF-8/TW-CA for RhB (10 mg L^−1^).

We investigated the effect of the adsorption temperature and pH on the dye solution. The effect of temperature on RhB adsorption by the samples was investigated using a kinetic study at three different temperatures (Figure 5a and Table 2). The results reveal that there was no significant difference in the adsorption capacities (9.5, 10.1, and 10.0 mg g^−1^ at 293, 308, and 318 K) of ZIF-8/TW-CA at the three temperatures, implying that the adsorption temperature had no significant effect on the dye adsorption in the temperature range of 293–318 K. RhB is a zwitterion dye in which the ionic state depends on pH; therefore, RhB changes from a cation to zwitterion form when the pH exceeds 3.7 (pKa of RhB). Consequently, this can affect the electrostatic interaction with the adsorbent. As shown in Figure 5b, the removal efficiency of ZIF-8/TW-CA was maintained within the pH range of 3–11, suggesting that RhB molecules can interact with other bonds through various mechanisms, such as hydrophobic interactions (π–π bonds), hydrogen bonds, and cation–π bonds of ZIF-8/TW-CA, except electrostatic interactions [71]. 

Figure 5c shows the UV–VIS spectra of the RhB solution (10 mg L^−1^) after 24 h adsorption by ZIF-8/DW-CA and ZIF-8/DW-CA. The color of the RhB solution disappeared when ZIF-8/TW-CA was immersed, whereas the color remained in the case of ZIF-8/DW-CA, indicating the enhanced porosity of ZIF-8/TW-CA for dye adsorption. Compared to that of DW, the loading of ZIF-8, with a high surface area, on TW was higher, owing to the presence of carboxyl groups (Figure 4a). In addition, the nanoporous structure of the cell wall (Figure 2g) caused by TEMPO treatment could facilitate the efficient capture of dye molecules in the solution. These factors can increase the specific area and micro/mesopores of ZIF-8/TW-CA (Figure 4b and Table 1). Figure 5d describes the effect of the initial RhB concentration on the adsorption capacity of ZIF-8/TW-CA. In this study, we employed two well-known adsorption isotherm models (Langmuir and Freundlich) to evaluate the experimental results. The parameters of the Langmuir and Freundlich isotherms are summarized in Table S1.

The Freundlich model was more suitable for the analysis of the adsorption isotherm, and it exhibited a high correlation coefficient (R^2^ = 0.99). The maximum adsorption capacities (q_max_) of balsa wood, ZIF-8/DW-CA, and ZIF-8/TW-CA were 43.15, 119.4, and 169.4 mg g^−1^, respectively. Particularly, the adsorption capacity of ZIF-8/TW-CA for RhB was approximately 1.4 times higher than that of ZIF-8/DW-CA. Importantly, the ZIF-8/TW-CA exhibited a good adsorption capacity (169.4 mg/g) for RhB comparable to previously reported values of other adsorbents, including biomass-based activated carbons (35.7 to 161.8 mg/g) (Table 3) [71,72,73,74,75,76,77,78,79,80,81,82]. The kinetic adsorption of ZIF-8/DW-CA and ZIF-8/TW-CA was observed for 24 h (Figure 5e and Table 2), and the result revealed that the pseudo-second-order kinetic model (R^2^ = 0.99) for chemical adsorption was more suitable for explaining the experimental data. A recycling test was conducted to investigate the reusability of ZIF-8/TW-CA. The used adsorbent was washed several times with a mixed solution of DIW and ethanol (1:1, 60 °C) until no dye was released, and then immersed in a fresh RhB solution (10 mg L^−1^). As shown in Figure 5f, even after three adsorption rounds, there was only a slight difference in the removal efficiency (96%) of ZIF-8/TW-CA. This result demonstrates the good reusability of ZIF-8/TW-CA via a simple desorption method. Porous materials with high porosity and a good affinity for hydrophobic solvents can also be used for the removal of organic solvents from polluted water. Lastly, we tested the ability of ZIF-8/TW-CA for the removal of organic solvents (Figure S6), and the results revealed that it took just 3 and 15 s to remove n-hexane and chloroform from the surface of water and underwater, respectively. These results demonstrate the good affinity of ZIF-8/TW-CA for hydrophobic organic solvents and its usefulness for the removal of organic solvents from polluted water. Considering the cost-effectiveness, renewability, and eco-friendliness of the ZIF-8/wood-based CA, these functional wood-based nanocomposites can serve as a promising adsorbent platform for various practical applications.

## 4. Conclusions

In this study, we developed a highly porous, heteroatom (O, N)-doped CA based on ZIF-8 and wood via TEMPO oxidation, in situ synthesis, and carbonization/activation processes. The results demonstrate that the introduction of carboxyl groups through TEMPO oxidation enabled a high ZIF-8 loading content. The proposed ZIF-8/TW-CA exhibited a 3D hierarchical porous structure, a high specific area, and an excellent adsorption capacity toward organic pollutants. These results can be attributed to the successful combination of the natural wood template with an ordered open-channel structure and the microporous ZIF-8 nanocrystals. The ZIF-8/TW-CA exhibited a high specific surface area of 785.8 m^2^ g^−1^ and a high adsorption capacity of 169.4 mg g^−1^ for RhB. During the adsorption–desorption cycle experiments, ZIF-8/TW-CA retained approximately 96% of its initial adsorption capacity after three cycles. The findings of this study will provide insight into the development of functional wood-based nanocomposites.

## Figures and Tables

**Table 1:** BET surface area (S_BET_), average pore volume (V_m_), and pore-size distribution of the samples.

Samples	S_BET_ (m^2^ g^−1^)	V_m_ (cm^3^ g^−1^)	Pore Size (nm)
Balsa wood	1.5	0.02	46.0
ZIF-8/DW-CA	662.1	0.30	1.9
ZIF-8/TW-CA	785.8	0.46	2.4

**Table 2:** Adsorption kinetic parameters for RhB on ZIF-8/TW-CA at three different temperatures (293, 308, and 318 K).

Samples	Pseudo-First-Order			Pseudo-Second-Order		
	q_t_ (mg/g)	K_1_	R^2^	q_t_ (mg/g)	K_1_	R^2^
ZIF-8/DW-CA-293 K	8.6	0.13	0.94	8.7	0.01	0.95
ZIF-8/TW-CA-293 K	9.2	0.20	0.96	9.5	0.02	0.98
ZIF-8/TW-CA-308 K	9.7	0.32	0.98	10.1	0.04	0.99
ZIF-8/TW-CA-318 K	9.6	0.30	0.99	10.0	0.03	0.99

**Table 3:** Comparison of the maximum adsorption capacity of various adsorbents for RhB.

Adsorbent	Adsorbent (mg)	Adsorbate (mL)	q_e_ (mg/g)	References
Sugar-based carbon	100	1000	123.5	[71]
RGO-Ni nanocomposite	0.5	50	65.3	[72]
Cal-ZIF-67/AC	20	100	46.2	[73]
Iron-pillared bentonite	10	20	98.6	[74]
MWCNT-COOH	50	25	42.7	[75]
Fe_3_O_4_/Humic acid	50	100	161.8	[76]
ZIF-8@ZIF-67	0.4	20	143.3	[77]
Carbon xerogel	20	50	132.0	[78]
Coffee-activated carbon	200	200	83.4	[79]
Sawdust-activated carbon	10	10	35.7	[80]
Lignocellulosic-activated carbon	2000	1000	39.98	[81]
ZnCl_2_-activated carbon	200	100	46.7	[82]
ZIF-8/TW-CA	10	10	169.4	This work

## Data Availability

Data can be made available on request.

## Supplementary Materials

The following supporting information can be downloaded at: https://www.mdpi.com/article/10.3390/nano13192695/s1, Table S1. Adsorption isotherm model parameters for RhB on balsa wood, TW-CA, ZIF-8/DW-CA, and ZIF-8/TW-CA; Figure S1. Photographs of (a) pristine wood, (b) delignified wood (DW), (c) TEMPO-oxidized wood (TW), (d) ZIF-8/TW-A, and (e) ZIF-8/TW-CA. Scale bar = 0.5 mm; Figure S2. (a) Lignin contents of balsa wood, delignified wood, and TEMPO wood after Klason lignin analysis. (b) Fourier transform infrared (FT-IR) spectra of balsa wood and delignified wood; Figure S3. X-ray diffraction (XRD) pattern of the (a) synthesized ZIF-8 and (b) ZIF-8/TW-CA; Figure S4. (a) Adsorption spectra of RhB dye at various concentrations (0.1–10 mg/L). (b) Calibration curve for the adsorption of RhB dye; Figure S5. The corresponding pore-size distributions of ZIF-8/DW-CA. Figure S6. Photograph of the removal of (a) *n*-hexane (stained with Sudan III) and (b) chloroform (stained with Sudan III) by ZIF-8/TW-CA.
