# Supplementary material for: Multiscale Porous Carbon Materials by In Situ Growth of Metal–Organic Framework in the Micro-Channel of Delignified Wood for High-Performance Water Purification
Source: Nanomaterials (Basel). 2023 Oct 3;13(19):2695. doi: 10.3390/nano13192695 (PMC10574260; doi:10.3390/nano13192695)
Supplement: Supplementary file 1 [file nanomaterials-13-02695-s001.zip › nanomaterials-2631474-supplementary.pdf]

Supporting Information

Multiscale Porous Carbon Materials by In-situ Growth of Metal-Organic Framework in the Micro-channel of Delignified Wood for High-Performance Water Purification

Youngho Jeon[1], Dabum Kim[1], Suji Lee[1], Kangyun Lee[1], Youngsang Ko[1], Goomin Kwon[1], Jisoo Park[2], Ung-Jin Kim[1], Sung Yeon Hwang[1], Jeonghun Kim[2,*], Jungmok You[1,*]

[1] Department of Plant & Environmental New Resources and Institute of Graduate School of Green-Bio Science, Kyung Hee University, 1732 Deogyeong-daero, Giheung-gu, Yongin-si, Gyeonggi-do, 17104, Republic of Korea
[2] Department of Chemical and Biomolecular Engineering, Yonsei University, 50 Yonsei-ro, Seodaemun-gu, Seoul, 0372, Republic of Korea

***Corresponding Author:**

J. Kim, E-mail: jhkim03@yonsei.ac.kr

J. You, E-mail: jmyou@khu.ac.kr

Table S1. Adsorption isotherm model parameters for RhB on balsa wood, TW-CA, ZIF-8/DW-CA, and ZIF-8/TW-CA.

Samples	Experimental q_{max} (mg/g)	Langmuir			Freundlich		
		q_{max} (mg/g)	K_L	R^2	n	K_F	R^2
Balsa wood	43.15	51.06	0.0071	0.95	2.5	3.05	0.91
ZIF-8/DW-CA	119.4	129.64	0.0076	0.94	2.69	9.637	0.98
ZIF-8/TW-CA	169.4	173.4	0.0102	0.92	2.85	15.876	0.99

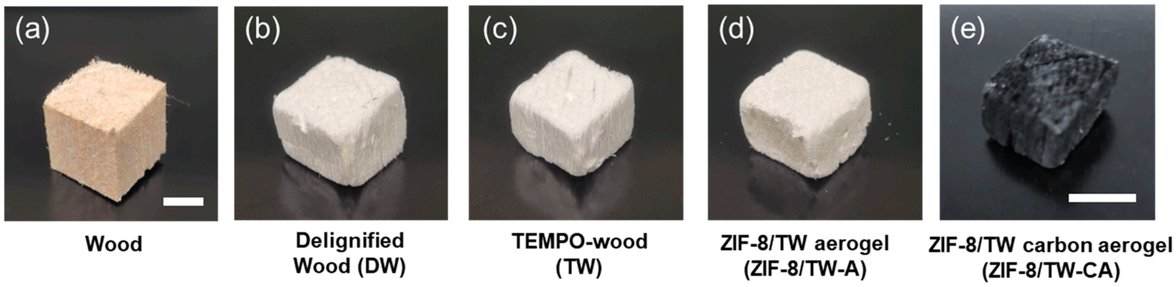

Figure S1. Photographs of (a) pristine wood, (b) delignified wood (DW), (c) TW, (d) ZIF-8/TW-A, and (e) ZIF-8/TW-CA. Scale bar = 0.5 mm

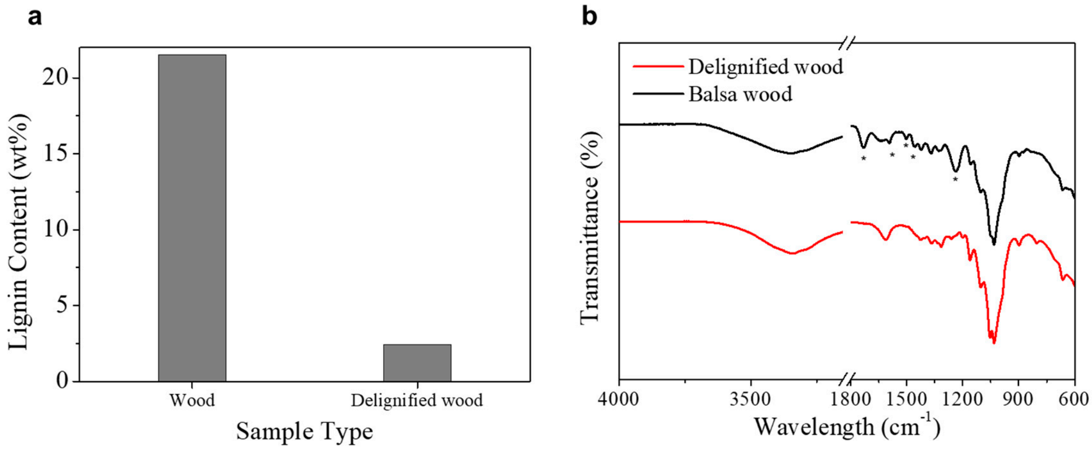

Figure S2. (a) Lignin contents of balsa wood, delignified wood and TEMPO wood after Klason lignin analysis. (b) FT-IR spectra of balsa wood and delignified wood.

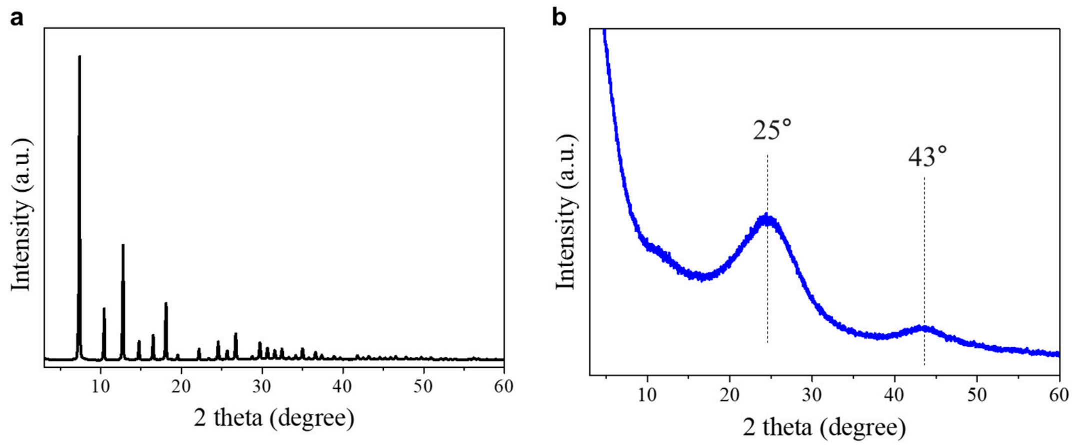

Figure S3. XRD pattern obtained from (a) synthesized ZIF-8 and (b) ZIF-8/TW-CA.

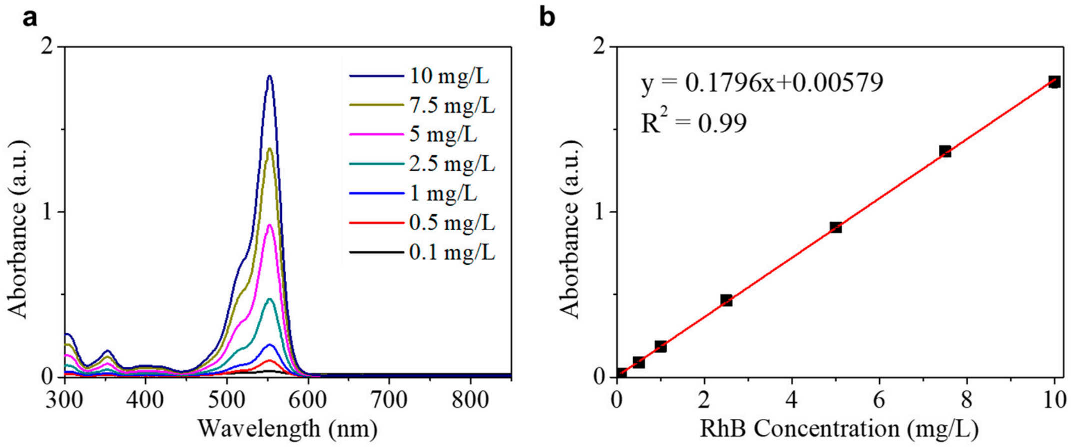

Figure S4. (a) Adsorption spectra of RhB dye at diverse concentrations (0.1–10 mg/L). (b) Calibration curve for the adsorption of RhB dye.

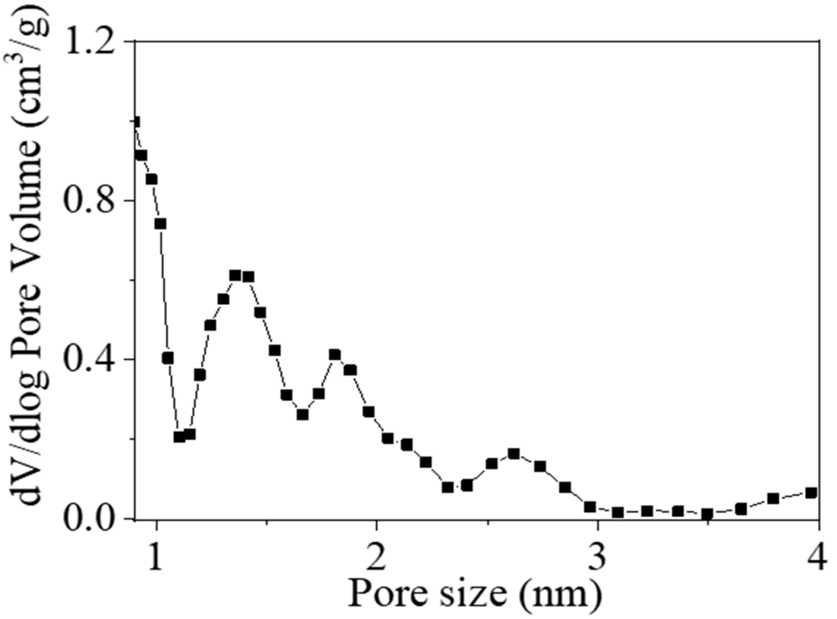

Figure S5. The corresponding pore-size distributions of ZIF-8/DW-CA.

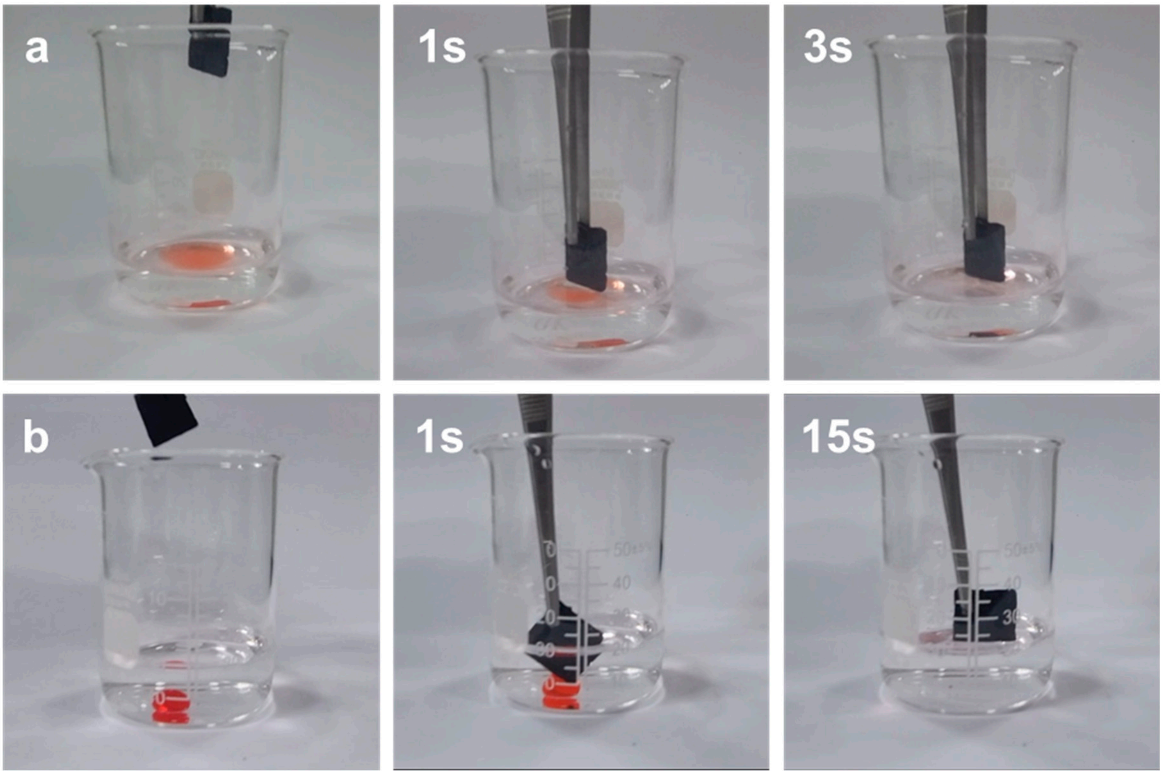

Figure S6. Photograph of the removal of (a) *n*-hexane (stained with Sudan III) and (b) chloroform (stained with Sudan III) by using ZIF-8/TW-CA.
